# Ovarian metastasis from lobular breast carcinoma: A case report with review of literature

**DOI:** 10.1016/j.radcr.2025.03.043

**Published:** 2025-04-05

**Authors:** Yasameen M. Mohammed, Zuhair D. Hammood, Abdulwahid M. Salih, Hadeel A. Yasseen, Soran H. Tahir, Sami S. Omar, Rebaz Haji Ali, Rawa M. Ali, Belan Mikaeil M. Radha, Ayman M. Mustafa, Fahmi H. Kakamad

**Affiliations:** a Department of Surgery, Tikrit Teaching Hospital, College of Medicine Street, Saladin, Iraq; b College of Medicine, University of Sulaimani, Madam Mitterrand Street, Sulaymaniyah, Kurdistan, Iraq; c Scientific Affairs Department, Smart Health Tower, Madam Mitterrand Street, Sulaymaniyah, Kurdistan, Iraq; d Kscien Organization for Scientific Research, Hamdi Street, Azadi Mall, Sulaymaniyah, Kurdistan, Iraq; e Department of Oncology, Rizgary Oncology Center, Peshawa Qazi Street, Erbil, Kurdistan, Iraq; f Department of Oncology, Hiwa Cancer Hospital, Sulaymaniyah, Kurdistan, Iraq

**Keywords:** Breast cancer, Invasive lobular carcinoma, Ovarian metastasis, Primary ovarian cancer

## Abstract

Although breast cancer can metastasize to various sites, involvement of the ovaries is infrequent. The current study aims to report a case of ovarian cancer originating from the breast along with an in-depth analysis of the literature. A 58-year-old woman with a history of invasive lobular carcinoma underwent surgery followed by adjuvant chemo-radiotherapy. During regular follow-up, a suspicious ovarian mass was detected, later confirmed to be metastasis from the breast cancer. A review of 25 studies on Google Scholar and PubMed identified 7185 ovarian metastasis patients, with 1253 cases originating from breast cancer, accounting for approximately 17.4% of all metastatic ovarian cancers. Bilateral ovarian involvement was noted in 75% of cases, with right-side involvement in 5.7% and left-side in 19.3%. Only 13 studies documented menopausal status, showing 53.6% were premenopausal. Controversies persist in distinguishing primary ovarian cancer from metastasis using clinical signs, serum markers, and imaging. Metastasis of breast cancer to the ovaries is an uncommon event, even after utilizing various therapeutic approaches. Surgery is the treatment of choice for these cases. This case highlights the importance of long-term surveillance in breast cancer patients and emphasizes the role of surgery in managing ovarian metastases.

## Background

Breast cancer stands as the most prevalent form of cancer globally, with over 2.3 million new cases each year. It is a primary cause of female mortality worldwide, with over 685,000 deaths in 2020, as reported by the GLOBOCAN database [1]. Additionally, breast cancer frequently metastasizes to the lungs, brain, liver, and bones [2,3]. Ovarian metastasis, although less frequent in cases of breast cancer [3,4], presents unique challenges. Women diagnosed with breast cancer have 3–7 times higher chances of developing primary ovarian cancer (POC) compared to ovarian metastasis (OM) [3,5,6], with the duration between breast cancer diagnosis and the development of POC tends to be shorter than in cases of breast cancer metastasizing to the ovaries [6].

Various risk factors contribute to the development of both breast and ovarian cancers, including age, exogenous estrogen, low parity, and a family history of breast cancer due to mutations in the breast cancer genes BRCA1 and BRCA2 (6). Breast cancer spreading to the ovaries constitutes 3%-38% of all ovarian malignancies in different studies [3,5,7]. The incidence rate and pattern of spread of primary malignancies significantly influence the occurrence of metastatic ovarian tumors. As the annual number of women diagnosed with breast cancer rises, so does the number of women with ovarian metastasis [6,8]. Common primary sites for ovarian metastasis are reported to be the gastrointestinal tract (39%-73%) particularly colorectal cancer and gynecological organs (10%-20%) [6]. Although discrimination of POC from OM is difficult from a clinical perspective, imaging techniques like ultrasound can provide beneficial information, as metastatic lesions in the ovaries are smaller, have more solid components, are bilateral, and show substantial extra-ovarian spread [3,5,9]. Metastatic ovarian tumors are typically chemo- and radio-resistant; however, cytoreductive surgery has been shown to have beneficial effects on symptom relief and prognosis [3,6,8,10]. The current study aims to report a case of a female patient with ovarian metastasis originating from breast cancer, along with an in-depth analysis of the relevant literature.

## Case presentation

### Patient information

**Fig. 1:**
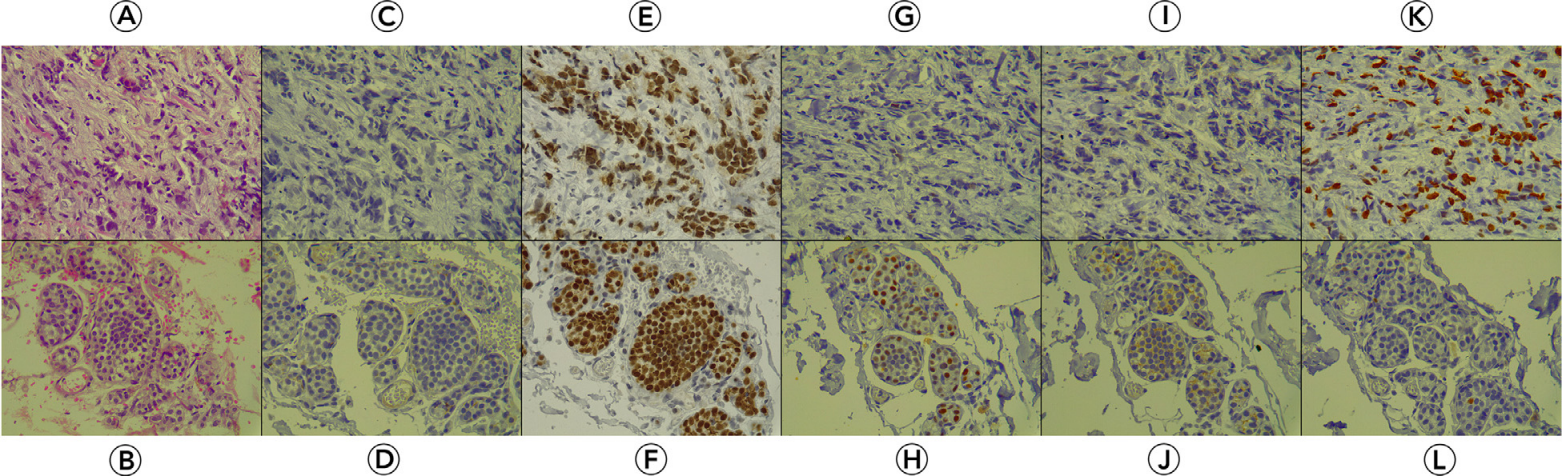
(A) The invasive breast tumor consists of large tumor cells arranged individually and in ribbons, characterized by indistinct cytoplasmic borders, lightly eosinophilic cytoplasm, and large, pleomorphic, hyperchromatic nuclei with irregular outlines, within a desmoplastic stroma. (B) The breast's in-situ component comprises expanded lobules with large, discohesive cells, featuring moderate lightly eosinophilic cytoplasm and uniform, round, hyperchromatic nuclei. (C-D) Both components show loss of membranous (E-cadherin staining. (E-F) Both components show nuclear staining of strong intensity for estrogen receptors. (G-H) Progesterone receptors show weak and patchy nuclear staining in the invasive component, while the in-situ component shows more diffuse and stronger staining. (I-J) The invasive component shows negative HER2/neu staining (score 0) while the in-situ component shows partial and weak membranous staining (score 1, also considered negative). (K-L) The invasive component has a high Ki-67 staining ratio (60%) while the in-situ component shows only scattered positive cells. [Hematoxylin and eosin (A-B), immunohistochemical antibodies with diaminobenzidine chromogen (C-L); original magnification x400 (A-L)].

A 58-year-old female patient, with no significant family history of breast or ovarian cancer, was diagnosed with left-sided breast cancer in 2019 after presenting with a palpable breast lump and nipple retraction. A core needle biopsy confirmed invasive lobular carcinoma. Staging investigations, including contrast-enhanced computed tomography and bone scintigraphy, revealed no distant metastases at the time of diagnosis. She subsequently underwent a modified radical mastectomy with axillary clearance, with histopathological examination revealing a T3N3M0 tumor stage due to multiple positive axillary lymph nodes. Immunohistochemical studies indicated positivity for estrogen (ER) and progesterone receptors (PR), while human epidermal growth factor receptor (HER2) was negative (Fig. 1). Subsequently, the patient received chemoradiotherapy and has since been maintained on hormonal therapy with an aromatase inhibitor, with regular follow-up.

### Diagnostic approach

**Fig. 2:**
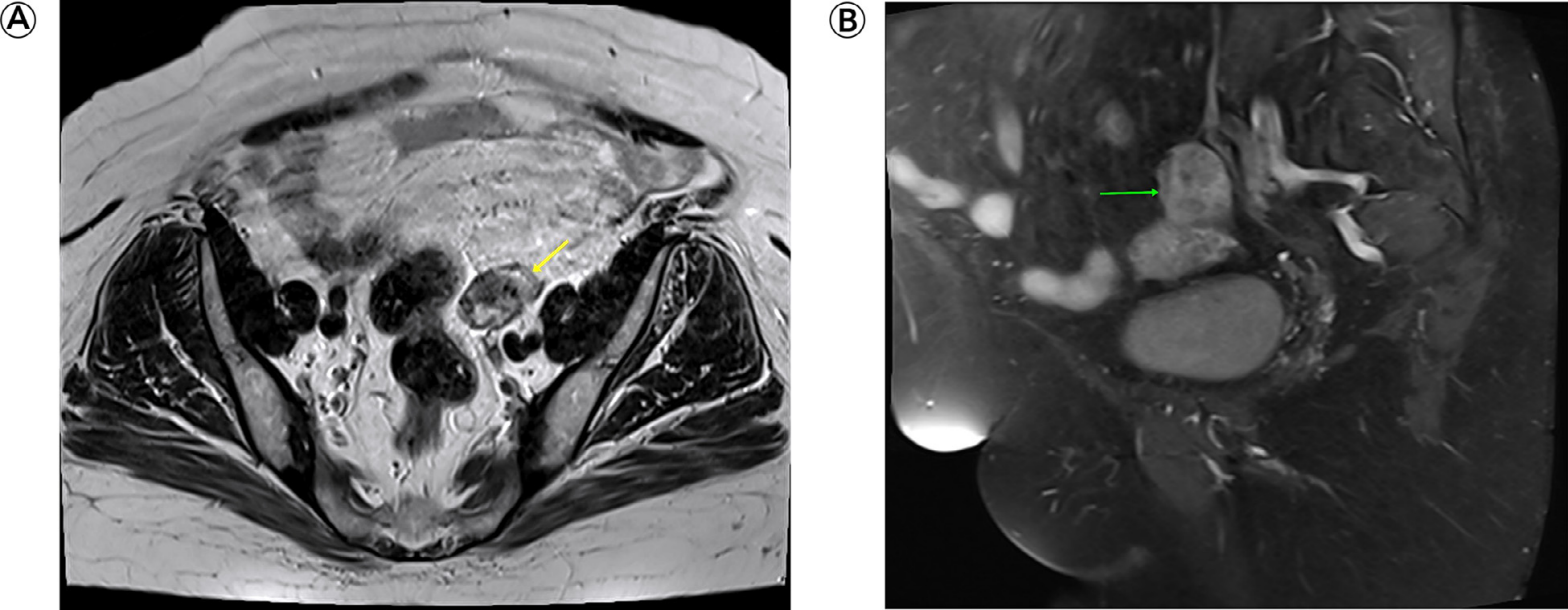
Pelvic MRI. (A) Axial T2 section shows a mass of heterogeneous intensity in the left ovary (yellow arrow) (B) Contrast-enhanced T1 fat suppression shows a heterogeneously enhancing mass (green arrow).

During routine follow-up, the patient underwent an abdominal ultrasound, which revealed a suspicious lesion in the left ovary, confirmed by a transvaginal ultrasound. Pelvic magnetic resonance imaging (MRI) scan revealed a 3.9 × 3.3 × 2.4 cm heterogeneously enhancing mass in the left ovary, suggestive of a malignant ovarian tumor, either metastatic or primary in origin (Fig. 2). Fluorodeoxyglucose positron emission (FDG PET) scan findings were inconclusive, suggesting a benign nature of the lesion.

Laboratory investigations revealed normal levels of tumor markers, including carbohydrate antigen 125 (CA125) (12 U/mL, normal <35 U/mL), carbohydrate antigen 15-3 (CA 15-3) (18 U/mL, normal < 30 U/mL), and cancer embryonic antigen (CEA) (2.1 ng/mL, normal < 3.0 ng/mL). The lack of significant elevation in these tumor markers, typically seen in primary ovarian cancers, made differentiation between primary and metastatic disease more challenging.

### Therapeutic approach

**Fig. 3:**
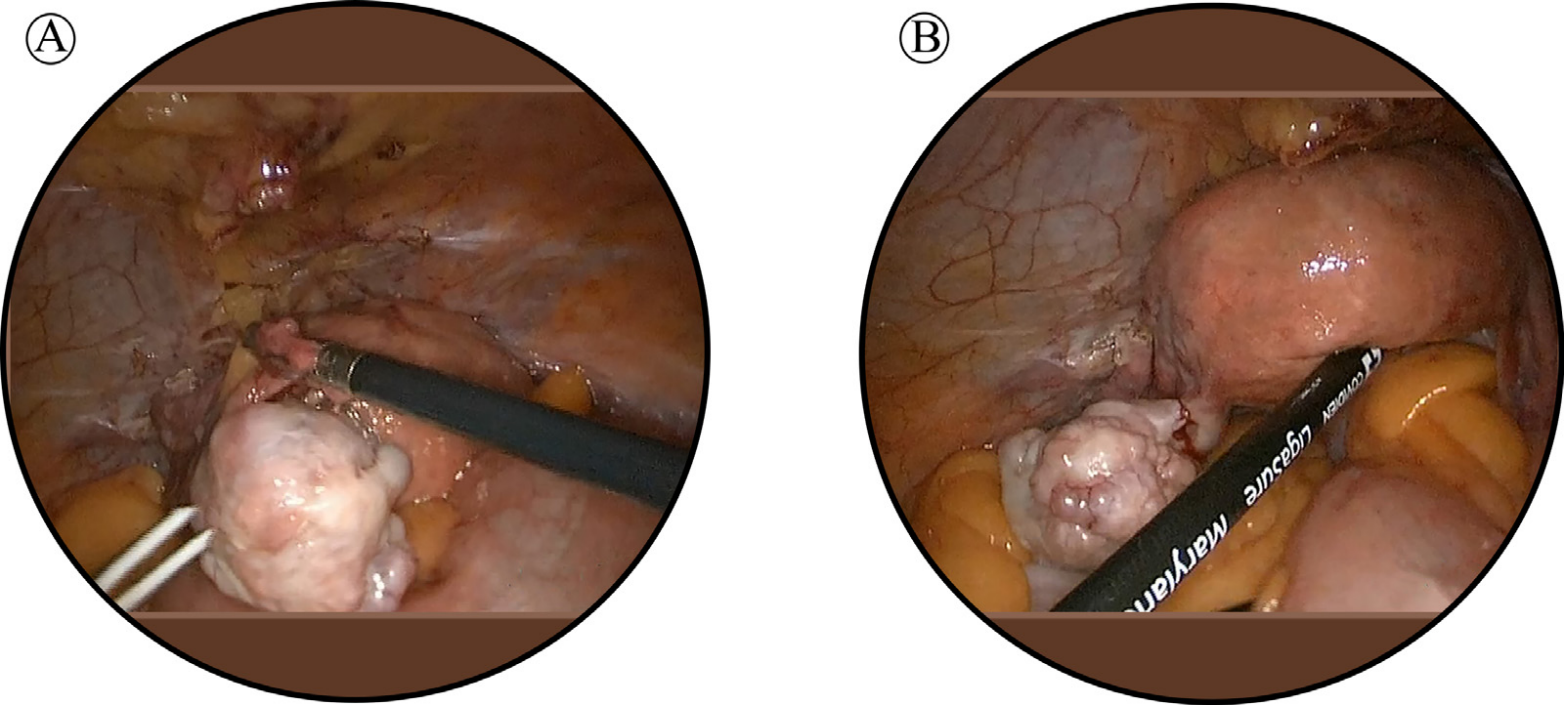
(A) Intraoperative view of the left ovarian tumor. (B) Panoramic view of the uterus with both ovaries and tubes.

**Fig. 4:**
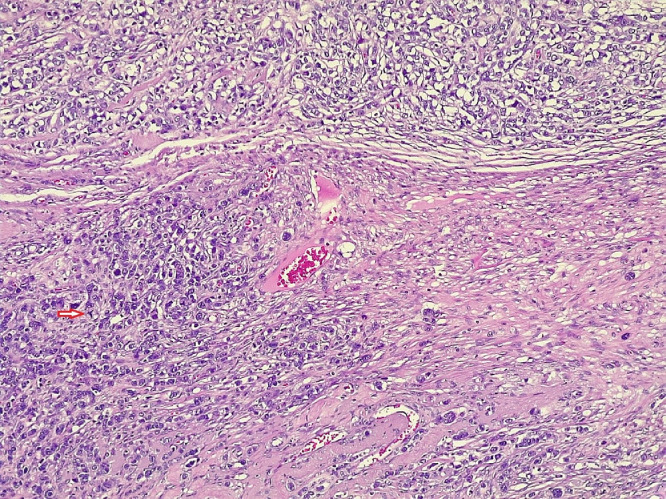
Histology of the left ovarian mass reveals highly pleomorphic tumor cells that have hyperchromatic nuclei with visible nucleoli and abnormal mitotic figures (red arrow), heavily infiltrating as discohesive sheets and singly into a desmoplastic stroma (H&E, x200).

**Fig. 5:**
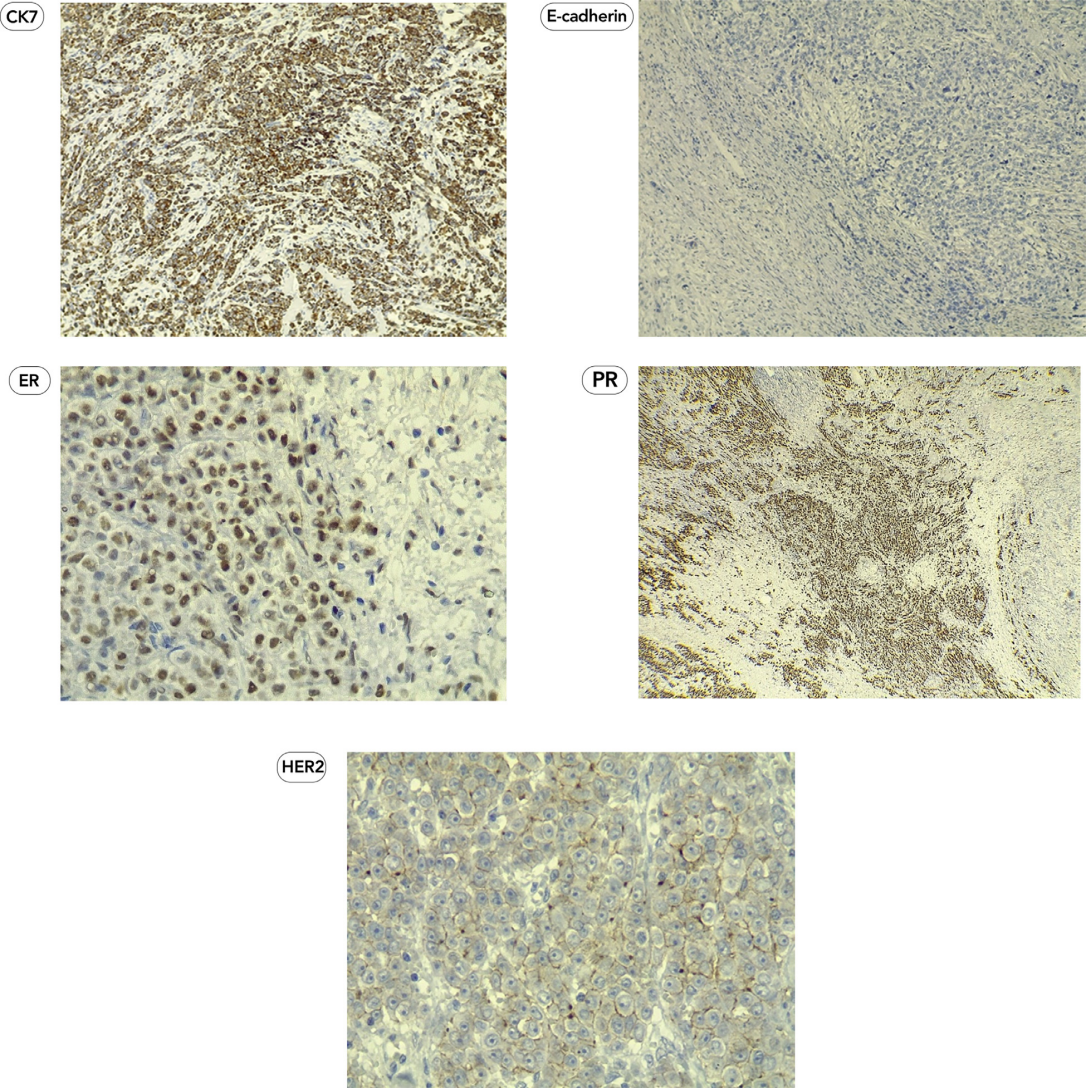
The tumor cells show strong cytoplasmic staining for CK7, negative staining for E-cadherin, positive nuclear staining for ER (score 6) and PR (score 6), and partial and weak cytoplasmic/membranous staining for Her-2 (score 1, considered negative).

Given the indeterminate nature of the ovarian lesion and the patient's history of hormone receptor-positive invasive lobular carcinoma, a laparoscopic hysterectomy with bilateral salpingo-oopherectomy was performed after detailed counseling and obtaining informed consent (Fig. 3). Intraoperatively, no peritoneal or omental disease deposits were observed. The postoperative course was uneventful, and the patient recovered well without complications. Histopathological examination of the resected ovarian tissue revealed a left ovarian mass (measuring 4 cm) comprising metastatic lobular carcinoma of breast origin (Fig. 4), confirmed through immunohistochemistry (IHC), establishing a clear link to the patient's prior breast cancer. The Allred scoring system was used for receptor quantification, with a score of 6 for ER and PR. The score is a combination of staining intensity score (0 = none, 1 = weak, 2 = moderate, 3 = strong) and positive tumor cell percentage score (0 = none, 1 = <1%, 2 = 1-10%, 3 = 11-33%, 4 = 34-66%, 5 = >66%) (Fig. 5). These findings established a definitive link to the patient's prior breast cancer.

### Follow-up and outcome

Following confirmation of ovarian metastasis, the patient was referred to the medical oncologist, who initiated fulvestrant injections and palbociclib tablets as a part of a standard endocrine-based treatment regimen. She continues to undergo regular follow-ups, with no evidence of further metastatic progression to date.

## Discussion

Breast cancer represents a widespread and significant global health challenge, being both frequently diagnosed and a leading cause of death [1,11,12]. Generally, the discovery of a new ovarian cyst in women with a history of breast cancer tends to be benign in nature [13,14]. Although these patients face an elevated risk of POC [5,14]. Secondary ovarian malignancies comprise 5–30% of all ovarian cancers [3,6,15,16]. Lee et al. reported that 13.6% of their ovarian malignancy patients had metastatic ovarian cancer, while Webb et al. found OM in 28% of the 1285 cases of malignant ovarian tumors they studied. Conversely, Yada-Hashimoto documented the rate of OM at 21.1% [8,17,18].

In a literature review of metastatic ovarian cancer originating from the breast, out of 7,185 OM patients, 1,253 were diagnosed with ovarian cancer of breast origin, constituting approximately 17.4% of all metastatic ovarian cancer cases. Notably, the patient in the current case was 58 years old, higher than the reported average age of 49.2 years old in the literature (Table 1).

**Table 1:** Summary of the reported literature on metastatic ovarian tumor of breast origin.

Author/References	Places/years	Metastatic ovarian tumors (No.)	Ovarian metastasis of breast origin (No./%)	Age (years)-average	Laterality of ovarian tumor			Menopausal state	
					Left (%)	Right (%)	Bilateral (%)	Pre- menopause (%)	Post- menopause (%)
Tserkezoglou et al. [5]	Greece-2006	36	9 (25%	54	22%	None	78%	None	None
Bigorie et al. [7]	France-2010	29	29 (100%)	48	76%	None	24%	62%	38%
Lee et al. [8]	Korea-2009	112	20 (1.8%)	46,8	None	None	54.50%	65.20%	34.80%
Pimentel et al. [10]	France-2016	28	28 (100%)	52	36%	None	64%	None	None
Yada-Hashimoto [18]	Japan-2003	64	9 (14.1%)	50.3	None	None	None	None	None
Peters et al. [21]	Netherlands-2017	2648	63 (2.3%)	37	40.40%	None	59.60%	None	None
Ayhan et al. [23]	Turkey-2005	154	35 (22.7%)	42.8	None	None	None	67%	33%
Naito et al. [24]	Korea-2012	1	1 (100%)	54	None	None	100%	None	100%
Alvarado/Cabrero et al. [25]	Mexico-2013	150	20 (13%)	51	None	None	57.30%	61.30%	38.70%
Bruls et al. [26]	Netherlands-2015	2312	330 (14.3%)	49.5	14.50%	21.50%	64%	None	None
Demopoulos et al. [27]	USA-1987	96	32 (33.3%)	54.8	25%	31.30%	43.70%	42.70%	57.30%
de Waal et al. [28]	Netherlands-2009	116	32 (27.6%)	49.5	None	None	69%	44.00%	39.70%
Kondi-Pafiti et al. [29]	Greece-2011	97	15 (15.5%)	55	22.70%	24.70%	52.60%	44.30%	55.70%
Moore et al. [30]	USA-2004	59	5 (8.2%)	55	20.30%	13.60%	66.10%	None	None
Fujii et al. [31]	Japan-2006	1	1 (100%)	34	None	None	100%	100%	None
Durga et al. [32]	India-2018	1	1 (100%)	33	None	None	100%	100%	None
Sal V et al. [33]	Turkey-2016	131	17 (13%)	50	None	None	None	None	None
Tingler et al. [34]	USA-2006	N	526 (74.7%)	60.3	None	None	None	None	None
Kim WY et al. [35]	Korea-2010	158	3 (1.9%)	43	None	None	None	None	None
Gavriilidis et al. [36]	Greece-2012	1	1 (100%)	53	None	None	100%	None	100%
Van Dam et al. [37]	Belgium-2007	1	1 (100%)	65	None	None	100%	None	100%
Ozer et al. [38]	Turkey-2012	68	5 (7.35%)	47.3	None	None	None	None	None
Turan et al. [39]	Turkey-2006	75	5 (6.7%)	44	15.40%	None	84.60%	None	None
Kawakubo et al. [40]	Japan-2010	1	1 (100%)	50	None	None	100%	None	100%
Gagnon et al. [41]	Canada-1989	165	64 (38%)	48.6	36%	None	64%	None	None

The precise mechanism of breast cancer metastasis to the ovaries remains unclear, with spread occurring through either lymphatic channels or blood vessels [3,16]. and noncoding RNAs have also been implicated in the metastatic process [3,19]. Metastasis is typically identified in premenopausal young women [3,7,20], With factors such as bilaterality [7], positive lymph nodes [10,21], and stage III-IV breast cancer (10) showing a positive correlation with ovarian metastasis. Additionally, lobular carcinoma of the breast is noted for its increased tendency toward ovarian metastasis [22].

The current case involves a postmenopausal woman diagnosed with unilateral (left) stage III breast cancer, characterized by positive lymph nodes (T3N3M0) and lobular histology. Among the studies reviewed in the literature regarding the laterality of ovarian tumors, bilateral involvement was observed in approximately 75% of cases, while right-side involvement occurred in only 5.7% of cases, and left-side involvement in roughly 19.3% of cases. Out of the 25 studies reviewed in the genuine literature [[5], [7], [8], [10], [18], [21], [23], [24], [25], [26], [27], [28], [29], [30], [31], [32], [33], [34], [35], [36], [37], [38], [39], [40], [41]], only 13 documented the menopausal status of the patients, with the majority (53.6%) being premenopausal (Table 1). However, controversies persist regarding hormone receptor status in metastatic breast cancer of the ovaries [7,13,42]. In a study conducted by Cerkauskaite D et al. involving 24 patients, most individuals exhibited positivity for both ER (87%) and PR receptors (91%) [6]. The current case also showed positive ER and PR, while HER2 was negative.

The identification of OM can be achieved through various methods, such as clinical presentation, serum tumor markers, imaging studies, and histopathological examination. The patient's medical history often serves as the first indicator of the diagnosis. Distinguishing OM from POC in cases of breast cancer can be challenging. Notably, OM may lack noticeable symptoms, with many women being diagnosed based solely on the presence of masses by imaging. Typically, these tumors are bilateral, small, and solid. However, some patients may experience symptoms like ascites, pelvic pain, gastrointestinal complaints, vaginal bleeding, and in certain cases, pseudo-Meigs syndrome. The primary symptom observed in a study by Ayhan et al. was related to the gastrointestinal system, while Eitan et al. identified abdominal distension as a prominent symptom. These findings suggest that the condition is often paucisymptomatic [7]. It is important to remember that these manifestations are not conclusively indicative of either POC or OM. The current case manifested no observable symptoms.

Serum tumor markers such as CA-125, CA-15-3, and CEA can be employed to differentiate OM from POC. One study showed that multiple, small, bilateral ovarian tumors with a mild elevation of CA-125 (<80 U/ml) and high elevation of CA-15-3 (>100 U/ml) are more likely to be OM, while the reverse is true for POC [5]. Furthermore, CEA shows an evident elevation in OM [24]. In the current case, all tumor markers (CA125, CA15-3, and CEA) were within the normal range.

Imaging tests are crucial for diagnosing, staging, and monitoring patients. Transvaginal ultrasound is particularly important when an ovarian mass is suspected, as it can preoperatively suggest metastatic ovarian tumors of breast origin based on specific sonographic characteristics [9]. These features include being small, solid, bilateral, and displaying high vascularization. In the current case, the ovarian lesion was first confirmed through transvaginal ultrasound.

Histopathological examination is the ‘gold standard’ method for diagnosing ovarian metastasis of breast origin, and this includes macroscopic, microscopic, and immunohistochemical analysis [16]. Histopathology of the current case revealed a left ovarian (4cm) mass comprising infiltrative, discohesive, and pleomorphic tumor cells within a desmoplastic stroma, confirmed immunohistochemically to be metastatic lobular carcinoma of the breast.

Various studies propose different methods for distinguishing between ovarian metastasis (OM) and primary ovarian cancer (POC). However, these studies often lack focus on the specific types of POC used in the differentiation process. When contemplating the potential origin of ovarian cancer from metastasis of breast cancer, attention must be directed toward particular ovarian cancer types that may exhibit similar presentations or histological features. These encompass serous carcinoma, mucinous carcinoma, endometrioid carcinoma, clear cell carcinoma, transitional cell carcinoma (Brenner Tumor), and cases of metastatic breast cancer affecting the ovaries [43].

Surgery is considered feasible for the treatment of both POC and OM [5]. Although it provides an acceptable survival benefit for POC, there are controversies about whether surgery prolongs survival in OM of breast origin [4,16]. It is the surgeon's decision whether to perform de-bulking surgery, simple bilateral adnexectomy, or just biopsies for diagnosis, especially in asymptomatic patients [10]. Many studies have shown acceptable results and a positive impact on prognosis with cytoreductive surgery in selected candidates [3,7]. Since a high percentage of patients with OM of breast origin are premenopausal and show positive hormone receptor status, bilateral oophorectomy has been utilized as a surgical option [7]. The current case underwent laparoscopic hysterectomy with bilateral saplingo-oopherectomy, and the operation ended without any complications.

## Conclusion

Histopathological examination is the gold standard method for the diagnosis of OM. Laparoscopic hysterectomy serves as a definitive surgical option for the management of OM of breast origin.

## Consent for publication

Not applicable.

## Availability of data and material

All data and materials are kept by the first and corresponding authors.

## Patient consent

Consent has been obtained from the patient.
